# Breathlessness With Pulmonary Metastases: A Multimodal Approach

**Published:** 2013-11-01

**Authors:** Jeannine M. Brant

**Affiliations:** From Billings Clinic Cancer Center, Billings, Montana

## Abstract

**Case Study**

Sarah is a 58-year-old breast cancer survivor, social worker, and health-care administrator at a long-term care facility. She lives with her husband and enjoys gardening and reading. She has two grown children and three grandchildren who live approximately 180 miles away.

**SECOND CANCER DIAGNOSIS**

One morning while showering, Sarah detected a painless quarter-sized lump on her inner thigh. While she thought it was unusual, she felt it would probably go away. One month later, she felt the lump again; she thought that it had grown, so she scheduled a visit with her primary care physician. A CT scan revealed a 6.2-cm soft-tissue mass in the left groin. She was referred to an oncologic surgeon and underwent an excision of the groin mass. Pathology revealed a grade 3 malignant melanoma. She was later tested and found to have BRAF-negative status. Following her recovery from surgery, Sarah was further evaluated with an MRI scan of the brain, which was negative, and a PET scan, which revealed two nodules in the left lung.

As Sarah had attended a cancer support group during her breast cancer treatment in the past, she decided to go back to the group when she learned of her melanoma diagnosis. While the treatment options for her lung lesions included interleukin-2, ipilimumab (Yervoy), temozolomide, dacarbazine, a clinical trial, or radiosurgery, Sarah's oncologist felt that ipilimumab or radiosurgery would be the best course of action. She shared with her support group that she was ambivalent about this decision, as she had experienced profound fatigue and nausea with chemotherapy during her past treatment for breast cancer. She eventually opted to undergo stereotactic radiosurgery.

**DISEASE RECURRENCE**

After the radiosurgery, Sarah was followed every 2 months. She complained of shortness of breath about 2 weeks prior to each follow-up visit. Each time her chest x-ray was normal, and she eventually believed that her breathlessness was anxiety-related. Unfortunately, Sarah’s 1-year follow-up exam revealed a 2 cm × 3 cm mass in her left lung, for which she had a surgical wedge resection. Her complaints of shortness of breath increased following the surgery and occurred most often with anxiety, heat, and gardening activities, especially when she needed to bend over. Sarah also complained of a burning "pins and needles" sensation at the surgical chest wall site that was bothersome and would wake her up at night.

Sarah met with the nurse practitioner in the symptom management clinic to discuss her concerns. Upon physical examination, observable signs of breathlessness were lacking, and oxygen saturation remained stable at 94%, but Sarah rated her breathlessness as 7 on the 0 to 10 Borg scale. The nurse practitioner prescribed duloxetine to help manage the surgical site neuropathic pain and to assist with anxiety, which in turn could possibly improve Sarah’s breathlessness. Several nonpharmacologic modalities for breathlessness were also recommended: using a fan directed toward her face, working in the garden in the early morning when the weather is cooler, gardening in containers that are at eye level to avoid the need to bend down, and performing relaxation exercises with pursed lip breathing to relieve anxiety-provoked breathlessness. One month later, Sarah reported relief of her anxiety; she stated that the fan directed toward her face helped most when she started to feel "air hungry." She rated her breathlessness at 4/10 on the Borg scale.

**SECOND RECURRENCE: MULTIPLE PULMONARY NODULES**

Sarah’s chest x-rays remained clear for 6 months, but she developed a chronic cough shortly before the 9-month exam. An x-ray revealed several bilateral lung lesions and growth in the area of the previously resected lung nodule. Systemic therapy was recommended, and she underwent two cycles of ipilimumab. Sarah’s cough and breathlessness worsened, she developed colitis, and she decided to stop therapy after the third cycle. In addition, her coughing spells triggered bronchospasms that resulted in severe anxiety, panic attacks, and air hunger. She rated her breathlessness at 10/10 on the Borg scale during these episodes. She found communication difficult due to the cough and began to isolate herself. She continued to attend the support group weekly but had difficulty participating in conversation due to her cough.

Sarah was seen in the symptom management clinic every 2 weeks or more often as needed. No acute distress was present at the beginning of each visit, but when Sarah began to talk about her symptoms and fear of dying, her shortness of breath and anxiety increased. The symptom management nurse practitioner treated the suspected underlying cause of the breathlessness and prescribed oral lorazepam (0.5 to 1 mg every 6 hours) for anxiety and codeine cough syrup for the cough. Opioids were initiated for chest wall pain and to control the breathlessness. Controlled-release oxycodone was started at 10 mg every 12 hours with a breakthrough pain (BTP) dose of 5 mg every 2 hours as needed for breathlessness or pain. Sarah noted improvement in her symptoms and reported a Borg scale rating of 5/10. Oxygen therapy was attempted, but subjective improvement in Sarah’s breathlessness was lacking.

**END OF LIFE**

Sarah’s disease progressed to the liver, and she began experiencing more notable signs of breathlessness: nasal flaring, tachycardia, and restlessness. Opioid doses were titrated over the course of 3 months to oxycodone (40 mg every 12 hours) with a BTP dose of 10 to 15 mg every 2 hours as needed, but her breathlessness caused significant distress, which she rated 8/10. The oxycodone was rotated to IV morphine continuous infusion with patient-controlled analgesia (PCA) that was delivered through her implantable port. This combination allowed Sarah to depress the PCA as needed and achieve immediate control of her dyspneic episodes. Oral lorazepam was also continued as needed.

Sarah’s daughter moved home to take care of her mother, and hospice became involved for end-of-life care. As Sarah became less responsive, nurses maintained doses of morphine for control of pain and breathlessness and used a respiratory distress observation scale to assess for breathlessness since Sarah could no longer self-report. A bolus PCA dose of morphine was administered by Sarah’s daughter if her mother appeared to be in distress. Sarah died peacefully in her home without signs of distress.

Breathlessness, also known as shortness of breath, dyspnea, or air hunger, is a dreaded symptom for patients with cancer, especially for those at the end of life. Breathlessness contributes significantly to suffering and poor quality of life and has been associated with a poor prognosis (Cuervo Pinna, Mota Vargas, Redondo Moralo, Sánchez Correas, & Pera Blanco, 2009; Suh et al., 2010; Trajkovic-Vidakovic, de Graeff, Voest, & Teunissen, 2012). Activities of daily living, endurance, exercise, and comfort are also compromised as the patient is focused on the symptom. Breathlessness is often associated with other symptoms such as fear, anxiety, depression, restlessness, and insomnia. One study found it clustered with pain and numbness (Yamagishi, Morita, Miyashita, & Kimura, 2009).

According to the American Thoracic Society, breathlessness is defined as "a subjective experience of breathing discomfort that consists of qualitatively distinct sensations that vary in intensity. The experience derives from multiple physiological, psychological, social, and environmental factors and may induce secondary physiological and behavioral responses" (American Thoracic Society, 1999). This definition takes into account the complex interplay of factors involved in breathlessness. An estimated 15% to 55% of patients with cancer have breathlessness at diagnosis, and 18% to 79% experience it at the end of life (Ben-Aharon, Gafter-Gvili, Paul, Leibovici, & Stemmer, 2008; DiSalvo, Joyce, Tyson, Culkin, & Mackay, 2008; Pantilat, O’Riordan, Dibble, & Landefeld, 2012; Shumway, Wilson, Howard, Parker, & Eliasson, 2008). Like pain, breathlessness is a subjective symptom: It exists whenever the person experiencing it says it does, and it may or may not be correlated with objective assessments such as oxygen saturation (Tice, 2006).

## Etiology of Breathlessness in Patients With Cancer

Breathlessness is often multifactorial and may or may not be directly related to the cancer itself. Cancer treatment can be a contributing factor to breathlessness. For example, chemotherapy-induced pulmonary fibrosis related to bleomycin toxicity or cardiomyopathy secondary to anthracyclines can cause breathlessness. And finally, breathlessness may be unrelated to the cancer. Patients with comorbidities such as obesity, chronic obstructive pulmonary disease (COPD), and congestive heart failure (CHF) may present with breathlessness any time during the cancer illness (Booth, Moosavi, & Higginson, 2008).

The pathophysiology of breathlessness is dependent on the underlying disease process. Its mechanisms include increased mechanical loading on inspiratory muscles, airflow limitation, inspiratory muscle weakness, or increased ventilatory demand. Although some patients have breathlessness at rest and during activity, identified triggers of dyspneic episodes include exertion, orthopnea, secretions, laryngospasms, and anxiety (Edwards Hood & Harwood, 2004). Anxiety was a trigger of breathlessness for Sarah, as were cough and laryngospasm.

Clinicians should attempt to determine the underlying cause of the breathlessness, but lack of findings should not discount symptomatic distress. Oxygen saturation, arterial blood gases, pulmonary function tests, chest x-rays, and anemia levels can provide data about the underlying problem, but treatment should be individualized and based on symptomatic distress and not on the presence of objective findings (Nonoyama, Brooks, Guyatt, & Goldstein, 2007). Understanding the etiology of the breathlessness aids in managing the underlying cause and hence the symptom.

## Assessment

Assessment of breathlessness is the first step in detecting a problem. The American College of Physicians emphasizes that clinicians should regularly assess for breathlessness (American College of Physicians, 2008). Self-report is the gold standard for the presence of breathlessness, but other observable signs may also be present such as tachycardia, tachypnea, the use of accessory muscles, paradoxical breathing that involves inward abdominal movement during inspiration, grunting at the end of expiration, and nasal flaring (Campbell, Templin, & Walch, 2010). As with Sarah’s initial reports of breathlessness, objective signs may be lacking, but the patient’s subjective report of breathlessness is the most reliable indicator of its presence.

Several assessment tools for breathlessness exist. Two of the most commonly used tools include the Numeric Rating Scale (NRS) and the Modified Borg Scale (Eakin, Sassi-Dambron, Ries, & Kaplan, 1995). The NRS, being a single-item measure, may be beneficial for patients who cannot complete more extensive measures. The Modified Borg Scale, a NRS with descriptors, is another simple yet clinically useful tool. Both tools are presented in Table 1.

**Table 1:**
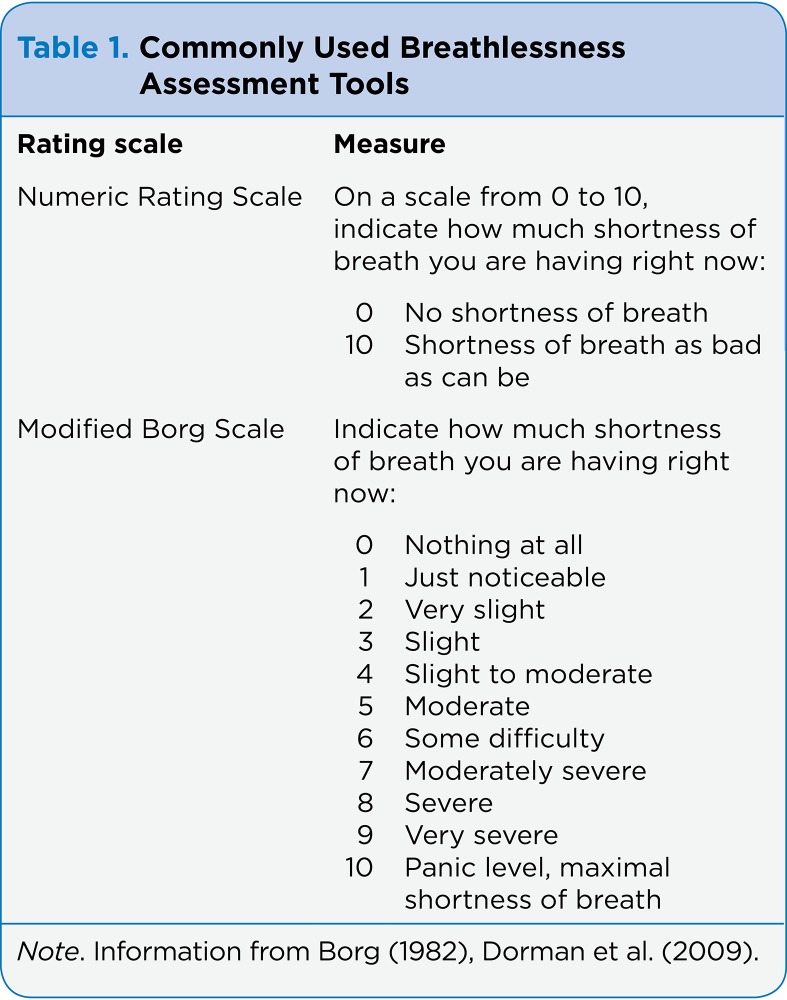
Table 1. Commonly Used Breathlessness Assessment Tools

For patients who are unable to communicate the presence or intensity of breathlessness, physical signs of distress should be used to assess and manage the discomfort (National Comprehensive Cancer Network [NCCN], 2011). One tool, the Respiratory Distress Observation Scale, is an eight-item assessment measure used for patients who are unable to self-report breathlessness (Campbell et al., 2010). Heart rate, respiratory rate, accessory muscle use, paradoxical breathing pattern, restlessness, grunting, nasal flaring, and fearful facial expressions are all considered in the nonverbal assessment. While internal consistency of the tool is questionable (á = 0.64), it may provide some guidance for patients unable to self-report breathlessness.

## Management

The management of breathlessness includes both treating the underlying cause and providing symptomatic relief. The NCCN provides some guidance within the palliative care guidelines for the treatment of breathlessness (NCCN, 2013). It is important to point out that interventions are based on life expectancy. For patients who have weeks, months, or years to live, strategies are aimed at treating the underlying cause and promoting comfort. For dying patients with a life expectancy estimated in days to weeks, relief of symptoms is the primary focus. A discussion of the strategies to manage breathlessness follows. Advanced practitioners should keep in mind that as life expectancy diminishes, interventions move from treating the underlying cause and employing ventilation and oxygen strategies toward comfort measures using opioids, benzodiazepines, and anticholinergic agents. For Sarah, the nurse practitioner began by treating the underlying cause of the dyspnea, but toward the end of life, relief-of-suffering interventions were the primary treatment modality.

**TREATING UNDERLYING CAUSES** 

As mentioned above, for patients who are not actively dying, strategies should be focused on treating the underlying cause of the breathlessness (see Table 2) while concurrently controlling symptomatic distress (see Table 3). Treatment can include therapies to shrink the tumor burden such as chemotherapy or radiation therapy. If breathlessness is related to pleural or peritoneal effusion, thoracentesis or paracentesis is an appropriate intervention. Unfortunately, the fluid can reaccumulate, leading to ongoing air hunger. For patients with recurrent pleural effusion, a small-bore tube can be inserted and secured into the pleural space to allow for long-term drainage and symptom relief (Kvale, Murthy, Taylor, Lee, & Nabors, 2009; Tice, 2006). Medications can also be useful in treating heart failure, pulmonary edema, COPD, and infection as underlying causes (American College of Physicians, 2008).

**Table 2:**
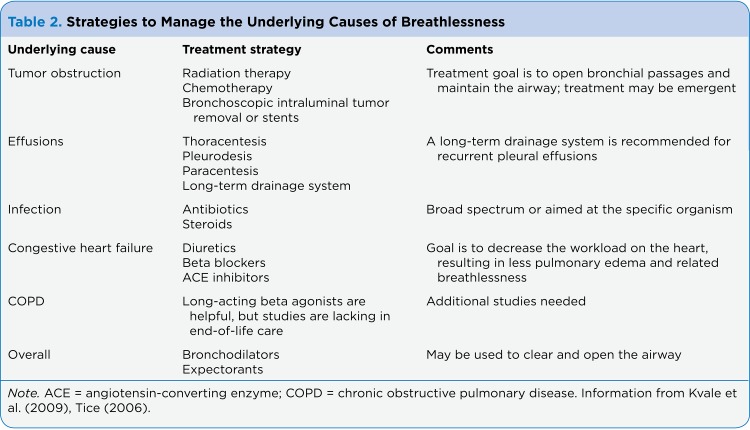
Table 2. Strategies to Manage the Underlying Causes of Breathlessness

**Table 3:**
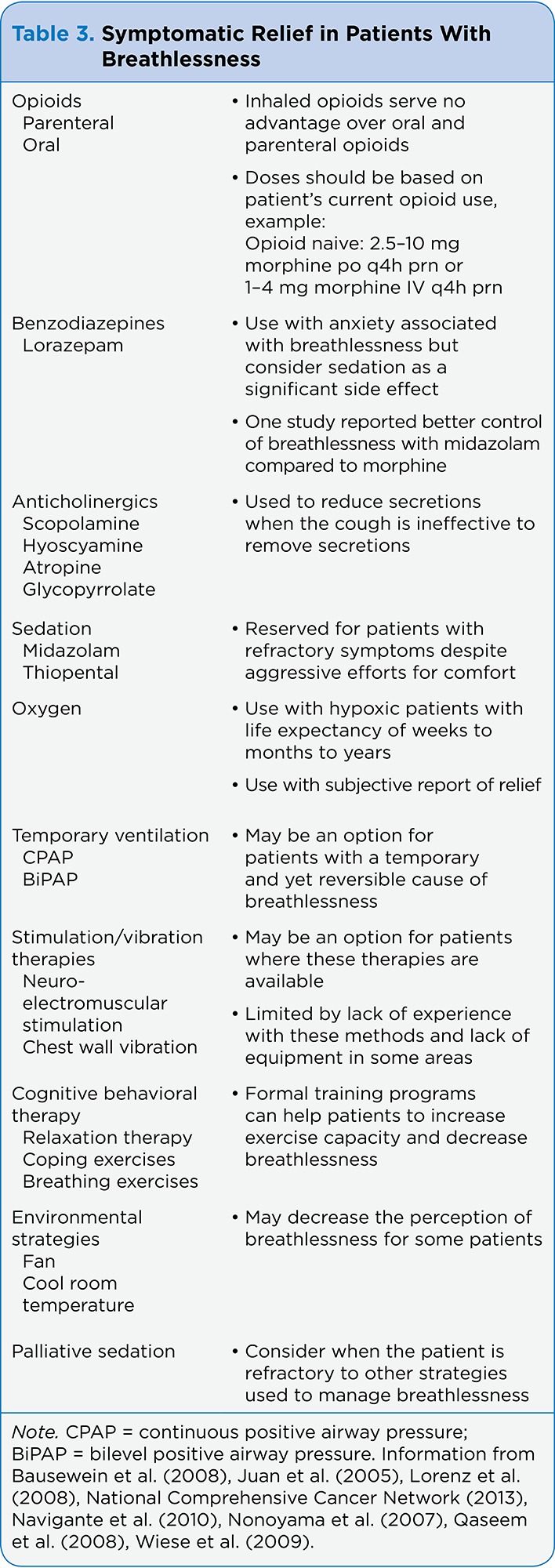
Table 3. Symptomatic Relief in Patients With Breathlessness

Some investigational treatments are worth noting. Tiotropium (Spiriva), an inhaled anticholinergic drug, was shown to improve airflow limitation, reduce hyperinflation, improve health-related quality of life, and increase exercise tolerance in patients with COPD (Ambrosino et al., 2008; Johansson, Hamberg, Westman, & Lindgren, 1999). Studies in cancer patients who may have associated COPD are lacking. In another study of interest, co-trimoxazole was used to manage breathlessness in patients with advanced fibrotic lung disease. A double-blind, randomized placebo-controlled trial of co-trimoxazole alone or in combination with oral prednisolone (n = 20) indicated that patients on the combined treatment arm had improved walk tests, increased oxygen saturation, and improvement of forced vital capacity (FVC). The mechanism of action is unknown but possibly related to antibacterial and antifungal mechanisms to decrease associated infection and immune-modulating properties, as the agent interacts with several cellular targets (Varney, Parnell, Salisbury, Ratnatheepan, & Tayar, 2008). Patients with cancer may experience fibrotic lung disease secondary to some chemotherapeutic agents, but again, studies in patients with cancer are lacking.

## Symptom Relief

**PHARMACOLOGIC STRATEGIES** 

Opioids are the mainstay therapy to relieve symptomatic distress. Systematic reviews, a meta-analysis, and NCCN guidelines support the use of oral or parenteral opioids for the relief of breathlessness. Nebulized opioids served no advantage over oral and parenteral options, although more studies are needed to confirm this finding (Ben-Aharon, Gafter-Gvili, Leibovici, & Stemmer, 2012; Juan et al., 2005; Lorenz et al., 2008; National Comprehensive Cancer Network, 2013; Qaseem et al., 2008). Opioids have also been used effectively in palliative care outpatients (n = 121). Breathlessness was satisfactorily ameliorated in 41% of patients, allowing patients to stay out of the hospital (Wiese, Barrels, Graf, & Hanekop, 2009).

Benzodiazepines such as lorazepam can be used for anxiety associated with breathlessness, although an analysis of several studies revealed a lack of evidence for benzodiazepine use in control of breathlessness for patients with cancer and COPD (Ben-Aharon et al., 2012; Simon, Higginson, Booth, Harding, & Bausewein, 2010). There was a nonsignificant trend toward a benefit, but side effects of drowsiness were substantial. The authors recommend using benzodiazepines as a second- or third-line treatment in patients with breathlessness, whereas the NCCN guidelines list benzodiazepines in the armamentarium to manage breathlessness associated with anxiety (NCCN, 2013). It is interesting to note that a recent randomized trial of morphine (n = 31) vs. midazolam (n = 32) in the management of cancer patients with breathlessness revealed that midazolam was better for the immediate and long-term relief of breathlessness; consequently, this agent may offer hope (Navigante, Castro, & Cerchietti, 2010).

**OXYGEN THERAPY** 

A lack of evidence exists to support the use of oxygen to control breathlessness in patients with cancer (Ben-Aharon et al., 2012; Lorenz et al., 2008; Nonoyama et al., 2007). However, oxygen may be recommended in patients who are hypoxic and have more than weeks to live as well as in all patients who report symptomatic relief with oxygen therapy (NCCN, 2011). In patients with COPD, a systematic review reported that 20 of 22 studies supported the use of oxygen with short-term exercise. Patients reported improved endurance and decreased breathlessness. Evidence was lacking to support oxygen at rest (Lorenz et al., 2008). As previously noted, oxygen did not provide relief of breathlessness for Sarah.

**NONPHARMACOLOGIC THERAPIES** 

A recent systematic review of 47 studies (N = 2,532 patients) examined evidence for nonpharmacologic therapies in controlling breathlessness (Bausewein, Booth, Gysels, & Higginson, 2008). High-level evidence exists for neuromuscular electrical stimulation (NMES) and chest wall vibration (CWV). To employ NMES, a stimulator is applied to the quadriceps muscle and stimulated for 20 minutes, 3 days per week. For CWV, vibrators are attached to the chest wall between the second and third intercostal spaces and 100-Hz vibrations are applied during inspiration for the upper vibrators and expiration for the lower vibrators.

Moderate-level evidence exists for the use of walking aids and breathing training. The review noted that additional studies are needed to determine whether distraction, relaxation, fans, counseling, case management, and psychotherapy were effective. Acupuncture had the lowest level of evidence. Some evidence supports the assertion that 4 weeks of pulmonary rehabilitation can result in a significant decrease in breathlessness (Lorenz et al., 2008).

As most of the studies in the systematic review were conducted in COPD patients, work is needed in this area to confirm these strategies in patients with cancer. In our case study, Sarah was open to trying relaxation exercises, and she reported benefit in trying to slow her breathing and release muscle tension. As noted in the case study, Sarah achieved some relief with a fan and relaxation and breathing training.

## Implications for Advanced Practitioners

Breathlessness in patients with cancer can occur at any time during the cancer trajectory, but it is more prevalent in patients with lung cancer and pulmonary metastases as well as in those at the end of life.

First, advanced practitioners have a primary responsibility to assess for breathlessness in all patients with cancer at each outpatient visit and during periods of hospitalization. Clinicians should keep in mind that observable signs of breathlessness may be absent; therefore, the most important component of the assessment is belief in the patient’s subjective report. Sarah lacked observable signs of breathlessness, and her oxygen saturation levels remained normal; however, she continued to experience distress related to her breathlessness. Toward the end of her life, when Sarah could no longer report breathlessness, hospice nurses continued the prescribed interventions and used an observation scale for ongoing assessment of her breathlessness.

Second, it is important to diagnose the etiology of the breathlessness so that strategies can be employed to treat the underlying cause. For some patients, eliminating the cause may provide total relief. The underlying cause of Sarah’s breathlessness may have been a decrease in ventilation capacity, cough, or anxiety. As a result, the nurse practitioner initially prescribed a suppressant to control the cough and an antidepressant and anxiolytic for the anxiety.

Finally, strategies should be focused on managing the sensation of breathlessness and its associated fear and anxiety. While the initial pharmacologic and nonpharmacologic interventions to treat the underlying cause gave Sarah some relief, they did not provide complete relief; therefore, oral opioids were prescribed, and they were successful in ameliorating Sarah’s breathlessness.

Overall, a multimodal approach is imperative in addressing patients with breathlessness. Managing its underlying cause as well as addressing the fear and the emotional component associated with the sensation of 
